# Serum Norepinephrine and Cholesterol Concentrations as Novel Diagnostic Biomarkers for Vitamin E Deficiency in Holstein Cows

**DOI:** 10.3390/ani15091333

**Published:** 2025-05-06

**Authors:** Yuxi Song, Xuejie Jiang, Yu Hao, Rui Sun, Yunlong Bai, Chuang Xu, Cheng Xia

**Affiliations:** 1 Heilongjiang Provincial Key Laboratory of Prevention and Control of Bovine Diseases, College of Animal Science and Veterinary Medicine, Heilongjiang Bayi Agricultural University, Daqing 163319, China; syxalz@163.com (Y.S.); jxj2862109645@163.com (X.J.); hy0314ai@163.com (Y.H.); a13936697304@163.com (R.S.); bai53626077@126.com (Y.B.); 2 College of Veterinary Medicine, China Agricultural University, Yuan Ming Yuan West Road No. 2, Haidian District, Beijing 100193, China

**Keywords:** dairy cows, vitamin E deficiency, GC-TOF-MS, LC-MS, metabolomics

## Abstract

Vitamin E deficiency (VED) represents a common micronutrient deficiency in dairy cows (DCs), leading to severe degenerative diseases, oxidative stress, immune dysfunction, and various health issues, ultimately causing significant economic losses for the global dairy sector. Accordingly, our objective was to explore the metabolic features of VED-afflicted cows by combining the untargeted gas chromatography-time-of-flight mass spectrometry (GC-TOF-MS) and targeted liquid chromatography-mass spectrometry (LC-MS) to identify effective serum VED biomarkers. Untargeted GC-TOF-MS analysis identified 31 differential metabolites (DMs): 20 were overexpressed and 11 were suppressed in the VED group compared to the healthy control group. These DMs were enriched in six major metabolic pathways: glycine, serine, and threonine; alanine, aspartate, and glutamate; cysteine and methionine; tyrosine; primary bile acid biosynthesis; and nitrogen metabolisms. These outcomes show that VED significantly disrupts amino acid/lipid/energy metabolism pathways in DCs. Further targeted LC-MS quantification revealed significant alterations in key metabolites, including increased levels of norepinephrine, glycine, cysteine, and L-glutamine, as well as a significant reduction in cholesterol concentrations. Binary logistic regression analysis identified norepinephrine and cholesterol as strong candidate biomarkers for VED. Receiver operating characteristic curve analysis established outstanding diagnostic accuracy for norepinephrine and cholesterol (for both *p* < 0.001, area under the curve = 0.980 and 0.990, correspondingly), with sensitivities and specificities of 90% and 100%, respectively. In conclusion, this study integrates untargeted and targeted metabolomics approaches to reveal VED-caused metabolic disruptions in DCs, particularly in amino acid/lipid/energy metabolism pathways. Norepinephrine and cholesterol were identified as highly accurate serum VED biomarkers with excellent diagnostic performance. Early detection and timely intervention using these biomarkers could promote disease treatment and cow health, as well as productivity, and decrease economic losses.

## Simple Summary

This study investigated metabolic alterations in dairy cows having vitamin E deficiency (VED) using combined gas chromatography-time-of-flight mass spectrometry and liquid chromatography-mass spectrometry metabolomics approaches. There were significant disruptions in amino acid/lipid/energy metabolism pathways, with 31 differential metabolites identified between VED and healthy control groups. Notably, norepinephrine and cholesterol were identified as promising serum biomarkers for VED diagnosis, showing excellent diagnostic accuracy with high sensitivity and specificity.

## 1. Introduction

Vitamin E deficiency (VED), a widespread micronutrient insufficiency in dairy cows (DCs) with a documented herd prevalence of 21% throughout the early lactation period (EL) [1], results in significant degenerative disorders, oxidative stress (OxS), immune dysfunction, and potential health issues, particularly lameness, left displaced abomasum, retained placenta, metritis, and mastitis, ultimately leading to substantial economic losses in the global dairy industry [2,3]. Vitamin E, known for its potent antioxidant properties, is a vital and intricate micronutrient essential for cows, with α-tocopherol being its primary bioactive form in circulation [4,5,6,7]. Moreover, VED can result in changes in α-tocopherol consumption, raised OxS and lipid peroxidation, and α-tocopherol transfer into colostrum around the time of calving [3]. Nevertheless, the physiological and metabolic mechanisms behind VED throughout the transition period (TP) in high-yielding DCs are still unclear.

The α-tocopherol plasma/serum concentrations in high-yielding DCs are progressively diminished during the prepartum phase, commencing multiple weeks prior to calving, attaining their nadir at calving, and persisting at diminished concentrations throughout the puerperal period (approximately 3–7 days) before gradually rising [3]. The National Research Council (NRC, 2001) guidelines [8] reported that α-tocopherol plasma/serum levels should exceed 3 μg/mL during the TP; falling below this threshold indicates a deficiency in α-tocopherol, known as VED. Thus, VED in adult DCs is characterized by a serum α-tocopherol concentration below 3 µg/mL [1,3,9,10].

Throughout the last 30 years, several investigations and reviews have explored VED-related disorder risk and the impact of α-tocopherol supplementation on the well-being and performance of both transition DCs and heifers. For instance, Haga et al. [3] highlighted VED as a significant risk factor for peripartum disorder and decreased performance in DCs during the TP. While supplementing α-tocopherol exceeding 3000 IU/day throughout the dry period is recognized as an essential strategy to prevent peripartum disease in high-yielding DCs, its efficacy in preventing VED around calving is not always guaranteed [3]. Certain proteins and metabolites may change at the onset of disease and persist at a consistently altered level throughout its progression. Regrettably, limited research has been conducted on the changed proteins and metabolites in VED-afflicted cows throughout TP. Lately, the proteomic and metabolomic profiles of VED-afflicted cows during the EL were analyzed in plasma and serum, respectively [9,10]. Nevertheless, the development of sensitive biomarkers for monitoring VED status in DCs continues to be a challenge, impeding progress in the field.

Recently, increasing utilization of gas chromatography-mass spectrometry (GC-MS) technology was presented in investigating metabolite variations between diseased and non-diseased states in cows. Studies have focused on conditions such as ketosis [11,12], subclinical mastitis [13,14], inactive ovaries [15,16], metritis [17,18,19], laminitis [20], and lameness [21], but not VED. The GC-MS is considered a robust and cost-effective analytical tool for metabolic phenotyping due to its high separation efficiency, high sensitivity, high accuracy, and low running cost [22,23]. Consequently, untargeted GC-MS analysis is a viable approach for obtaining more comprehensive and detailed insights into metabolite variations of VED-afflicted cows. Moreover, OxS arises from disrupted redox homeostasis resulting from inadequate antioxidant levels to counteract reactive oxygen species, potentially leading to metabolic changes [24]. As vitamin E is a potent antioxidant whose shortage can induce OxS, we assumed that VED may alter the metabolic profile of DCs during the TP. Therefore, the purpose of this investigation was to authenticate this hypothesis by applying untargeted gas chromatography-time-of-flight mass spectrometry (GC-TOF-MS) to analyze the serum metabolome in VED-afflicted cows compared to healthy cows. Furthermore, targeted liquid chromatography-mass spectrometry (LC-MS) with higher selectivity and sensitivity was utilized to quantify specific untargeted GC-TOF-MS results to identify effective serum VED biomarkers in DCs.

## 2. Materials and Methods

### 2.1. Animals and Diets

This investigation was performed with Holstein cows from a substantial, intensive cattle farm in central Heilongjiang Province, China, following the Ministry of Agriculture of China Veterinary Medical Ethics Committee Guidelines. Approval was obtained from Heilongjiang Bayi Agricultural University Animal Welfare and Research Ethics Committee (DWKJXY2023057; 1 August 2023). This study selected 27 clinically healthy multiparous Holstein DCs (singletons, aged 3.67 ± 0.89 years, with a parity of 2.67 ± 0.97, weighing 635.01 ± 26.86 kg, and yielding 37.37 ± 7.89 kg of milk daily, mean ± SD) from an intensive dairy farm in Heilongjiang, China, at day 21 postpartum. No DCs exhibited any clinical signs of disease (e.g., ketosis, mastitis, metritis, lameness) upon two veterinary examinations. According to serum α-tocopherol levels, cows were categorized into healthy control (HC, α-tocopherol > 4 μg/mL, *n* = 14) and VED (α-tocopherol < 3 μg/mL, *n* = 13) groups. Cows with intermediate values (3–4 μg/mL) were excluded before grouping, as described in Qian et al. and Song et al. [9,10]. Animals were housed in individual free-stall barns bedded with kiln-dried sawdust, equipped with fans and sprinklers, with access to a dirt exercise lot. They were provided with an EL total mixed rations (TMR) diet post-calving that adhered to the NRC (2001) guidelines [8], nourished thrice daily, and milked at 0600, 1400, and 2200 h. Table S1 presents the constituents and chemical content of the EL TMR. A specialized software (Afifarm version 5.3, Afimilk, Kibbutz Afikim, Israel) was deployed to document age, parity, weight, milk output, and dry matter intake (DMI). Two certified field veterinarians assessed the body condition score (BCS) utilizing a 5-point scale of 1–5, with 0.25-unit intervals [25].

### 2.2. Blood Collection

At day 21 postpartum, blood samples were obtained via the tail vein with vacuum blood collection tubes with no additives. The samples were protected from light exposure and permitted to clot for half an hour at a controlled room temperature (22–25 °C) before centrifugation (3500× *g*, 10 min) to separate the serum. The serum samples were maintained at −80 °C for subsequent biochemical, GC-TOF-MS, and ultraperformance LC-MS (UPLC-MS) analyses.

### 2.3. Serum Detection

Vitamin E (α-tocopherol) serum levels were assessed via high-performance liquid chromatography [26]. Serum β-hydroxybutyrate (BHB), non-esterified fatty acids (NEFA), and glucose levels were assessed utilizing commercial biochemical assay kits per protocols via the Mindray BS-830S fully automated biochemistry analyzer (Mindray Biomedical Electronics Co. Ltd., Shenzhen, China) at the Biotechnology Centre of Heilongjiang Bayi Agricultural University. The serum activities of total antioxidant capacity (T-AOC), superoxide dismutase (SOD), catalase, glutathione peroxidase (GSH-Px), glutathione (GSH), malondialdehyde (MDA), and hydroxyl radical (•OH) were quantified with commercial kits (Nanjing Jiancheng Bioengineering Institute Co., Ltd., Nanjing, China) per protocols.

### 2.4. Sample Preparation

Samples were prepared for GC-TOF-MS analysis [15] by first defrosting 27 serum samples at a controlled ambient temperature (22–25 °C), transferring 50 μL of each sample into a 1.5 mL tube, and adding 200 μL pre-chilled methanol and 5 μL internal standard (L-2-chlorophenylalanine, 1 mg/mL stock solution). The mixture was vortexed for 30 s, ultrasonicated in ice water for 10 min, centrifuged (12,000 rpm, 15 min, 4 °C), and transferred 180 μL of the supernatant to a new tube. Seeking the preparation of the quality control (QC) sample, 30 μL from each individual sample was collected and pooled. Following evaporation in a vacuum concentrator, 30 μL of methoxyamine hydrochloride (20 mg/mL in pyridine) was introduced and incubated at 80 °C for half an hour. Derivatization was performed by adding 40 μL of N,O-bis(trimethylsilyl)trifluoroacetamide (1% trimethylchlorosilane, *v*/*v*) and incubating at 70 °C for 1.5 h. Samples were gradually cooled to room temperature (22–25 °C), thereby introducing 5 μL of fatty acid methyl esters (dissolved in chloroform) to the QC sample. All samples were subsequently analyzed using GC-TOF-MS.

### 2.5. GC-TOF-MS Analysis

GC-TOF-MS analysis was carried out through an Agilent 7890B GC (Agilent Technologies, Santa Clara, CA, USA) equipped with a Pegasus HT TOF-MS (LECO Corporation, St. Joseph, MI, USA) and a DB-5MS capillary column (30 m × 250 μm × 0.25 μm; J&W Scientific, Folsom, CA, USA). Helium served as the carrier gas at 1 mL/min, with a 1 μL splitless injection. The column started at 50 °C for 1 min, then ramped to 310 °C at 20 °C/min and held for 6 min. The injector, transfer line, and ion source were set to 280, 280, and 250 °C, respectively. Mass spectra were acquired in electron impact mode (70 eV) with a source temperature of 250 °C, scanning from *m*/*z* 50–500 at 12.5 spectra/s after a 4.83 min solvent delay.

### 2.6. Data Preprocessing

Raw data analysis, including peak extraction, baseline correction, deconvolution, alignment, and integration, was performed via Chroma TOF software (version 4.3X, LECO Corporation, St. Joseph, MI, USA). Metabolite identification was conducted based on mass spectral and retention index matching against the LECO-Fiehn Rtx5 database [27]. Peaks detected in less than 50% of QC samples or exhibiting a relative standard deviation exceeding 30% in QC samples were excluded from further analysis [28].

### 2.7. Multivariate Statistical Analysis

Following data preprocessing through Pareto scaling, pattern recognition was executed utilizing SIMCA software (v15.0.2, Umetrics, Umea, Sweden), which included unsupervised principal component analysis (PCA) and supervised orthogonal projections to latent structures-discriminant analysis (OPLS-DA). The PCA was employed to assess intra-group clustering and inter-group differentiation while conducting OPLS-DA to elucidate inter-group disparities. The validation in OPLS-DA models was conducted by the analysis of variance in Y (R^2^Y) and predictive capability (Q^2^) via cross-validation and permutation testing, utilizing 200 iterations. Models were deemed stable and dependable when 1 > R^2^Y and Q^2^ ≥ 0.4 [29]. Furthermore, a Q^2^ intercept < 0.05 from the permutation test was employed to confirm the absence of overfitting [29], and univariate analysis was conducted, encompassing the Student’s *t*-test and fold change (FC) analysis.

### 2.8. Metabolite Identification and Pathway Analysis

Metabolites with significant differences were identified via OPLS-DA model-derived variable importance in projection (VIP) scores > 1, in conjunction with *p* < 0.05 from a Student’s *t*-test. The Euclidean distance matrix for the mathematical computations of the differential metabolites (DMs) was established for each set of analyses. The complete linkage method was employed to cluster the divergent metabolites, which were subsequently presented as a heatmap of hierarchical clustering. The Kyoto Encyclopedia of Genes and Genomes (KEGG) database (www.kegg.jp/kegg/pathway.html, last accessed on 31 December 2024) was utilized for the DMs associated with numerous metabolic pathways, presenting the results by a bubble plot.

### 2.9. Targeted Metabolomics Assays

Five DMs (norepinephrine, glycine, L-cysteine, L-glutamine, and cholesterol) were measured in serum through a UPLC-MS-validated methodology [30]. Briefly, 20 serum samples (10 samples/group) were first thawed at 4 °C, placing 10 μL of each sample in 1.5 mL centrifuge tubes. Then, 10 μL water, 5 μL internal standard, and 40 μL 0.1% isopropanol were added, vortexed, and centrifuged (10,000× *g*, 10 min, 4 °C). Then, 10 μL of the supernatant was transferred to a new 1.5 mL tube and derivatized with 70 μL of AccQ Tag borate buffer and 20 μL of AccQ Tag reagent (Waters, Kairos, Miliford, MA, USA). Following a 10-s vortex, the samples were heated to 55 °C for 10 min. Finally, 500 μL of ultrapure water was used to dilute the derivatized sample for LC-MS analyses. Metabolite analyses were conducted via a Waters ACQUITY UPLC I-Class system equipped with a Waters Xevo TQ-XS tandem quadrupole MS in positive ion mode. Separation was achieved on a Waters ACQUITY UPLC HSS T3 column (2.1 mm × 150 mm, 1.8 µm) maintained at 50 °C. The mobile phases comprised (A) 0.1% formic acid in water and (B) 0.1% formic acid in acetonitrile, with a flow rate of 0.5 mL/min and an injection volume of 5.0 μL. The gradient elution procedure was 0 min, 4% B; 0.5 min, 4% B; 2.5 min, 10% B; 5 min, 28% B; 6 min, 95% B; 7 min, 95% B; 7.1 min, 4% B; and 9 min, 4% B. The electrospray ionization conditions were set as follows: capillary voltage, 1.5 kV; con voltage, 20 V; desolvation temperature, 500 °C; source temperature, 150 °C; desolvation gas flow, 1000 L/h; and cone gas flow, 10 L/h. Quantification was performed in multiple reaction monitoring (MRM) mode.

### 2.10. Statistical Analysis

Cow was the experimental unit. Statistical analysis was carried out by means of IBM SPSS Statistics 26.0 for Windows (IBM Corp., Armonk, NY, USA). An independent samples *t*-test was employed to assess differences in clinical variables (age, parity, BCS, milk yield, and DMI), biochemical variables, and five targeted metabolites between the VED and HC groups. Data were presented as mean ± SD. Correlations between five targeted metabolites and VED were evaluated via Spearman rank correlation coefficients. Binary logistic regression models were produced for VED prediction. The receiver operating characteristics (ROC) curve was deployed to examine the significance of the logistic regression models. *p* < 0.05 and *p* < 0.01 indicated statistical significance.

## 3. Results

### 3.1. Background Attributes and Serum Biochemical Profiles

Table 1 shows that age, parity, and milk yield displayed no significant difference between both groups (*p* > 0.05). Meanwhile, the VED group exhibited significantly lower BCS and DMI than the HC group (*p* < 0.05). Unlike the HC group, VED-afflicted cows displayed increased serum MDA levels and reduced serum α-tocopherol (2.12 ± 0.66 vs. 6.73 ± 0.84 μg/mL), GSH, and T-AOC levels (*p* < 0.05).

### 3.2. DMs Identified by Untargeted GC-TOF-MS

Here, we analyzed the total ion chromatograms (TIC) of four QC samples, focusing on retention time (RT), peak intensity, and separation efficiency. The TIC overlap of QC samples was satisfactory, implying a robust approach with great reproducibility and stability. The sample TIC displayed that the peak morphology was preserved and that neighboring peaks were distinctly divided, demonstrating that the chromatographic and mass spectrometric conditions were appropriate for sample identification (Figure S1).

**Figure 1:**
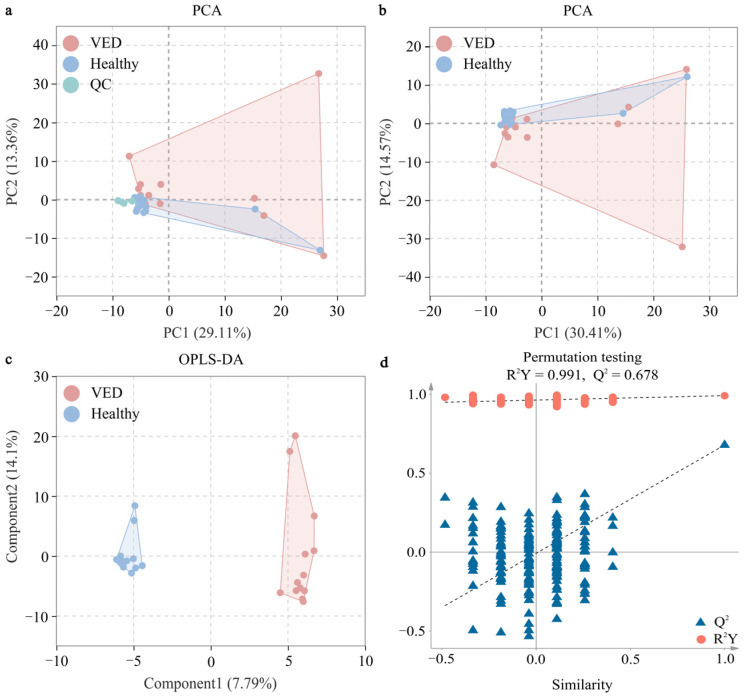
Multivariate statistical analyses of serum metabolites between both groups. (**a**) PCA score plot with and (**b**) without the QC samples. (**c**) OPLS-DA score plot. (**d**) Permutation test plot (200 permutation tests) for the OPLS-DA model. VED = vitamin E deficiency, QC = quality control, PCA = principal component analysis, OPLS-DA = orthogonal partial least squares discriminant analysis, R^2^Y = goodness-of-fit parameter, Q^2^ = predictive ability parameter.

The serum metabolic alterations in the VED group were examined against the HC group using multivariate statistics on the GC-TOF-MS data. Initially, we conducted an unsupervised PCA on the metabolomic profiling of 424 metabolites. The PCA score plot demonstrated that all QC samples congregated near the origin, signifying excellent repeatability (Figure 1a). The PCA score plot indicated a minimal distinction between both groups (Figure 1b). An OPLS-DA supervised model was implemented to attain optimal differentiation (R^2^Y = 0.991, Q^2^ = 0.678). Both R^2^Y and Q^2^ values were > 0.4, signifying a stable and dependable model (Figure 1c). A Q^2^ score of roughly 1 suggested that the OPLS-DA model exhibited strong predictive capability. The Q^2^ intercept values were < 0.05, signifying the absence of overfitting (Figure 1d).

**Figure 2:**
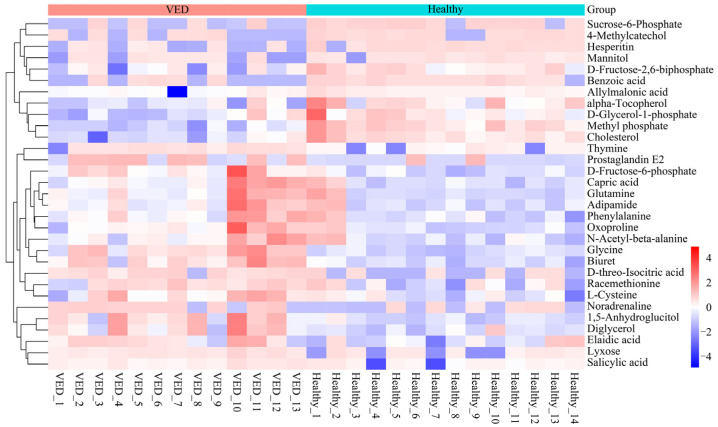
Heatmap visualization of the 31 DMs.

Variables having a VIP > 1 and *p* < 0.05 were classified as DMs. A total of 31 metabolites exhibiting differential regulation were detected in the VED group against the HCs, with 20 metabolites presenting overexpression and 11 metabolites presenting suppression (Table 2). The relative abundance of the 31 DMs was compared between the VED and HC groups, revealing a distinct clustering pattern on a heatmap (Figure 2). Notably, the relative quantification of α-tocopherol (FC = 0.40) in the serum metabolomic analysis was consistent with the trend observed in the conventional serum test.

### 3.3. Analysis and Identification of Key Metabolic Pathways

**Figure 3:**
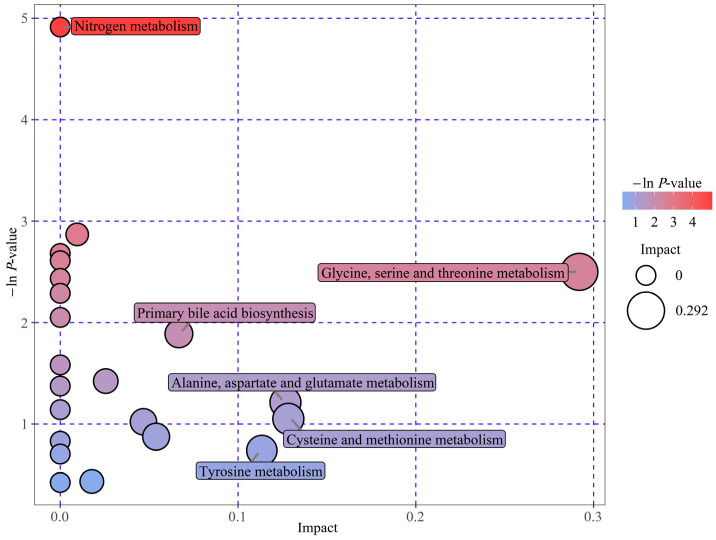
Enrichment bubble map: DM-related metabolic pathways identified in both groups. Bubble position and size on the abscissa donate pathway’s influence factor from topology analysis, with larger bubbles indicating greater influence. The position and color on the y-axis reflect the −ln(*p*-value) from enrichment analysis, with darker colors representing smaller *p*-values and more significant enrichment.

To elucidate the DM biological relevance, metabolic pathway enrichment analysis was performed by MetaboAnalyst 6.0 software (https://www.metaboanalyst.ca/, accessed on 31 December 2024). Herein, we deployed an interactive visualization approach to illustrate the key metabolic pathways related to the 31 DMs between VED and HC cows (Figure 3). A total of five pathway impacts greater than 0.05 were identified in the main metabolic pathways (Table 3). Among them, four biological modules were implicated in amino acid metabolism, specifically, glycine, serine, and threonine metabolism (impact = 0.29); alanine, aspartate, and glutamate metabolism (impact = 0.13); cysteine and methionine metabolism (impact = 0.13); and tyrosine metabolism (impact = 0.11). Additionally, one biological module was associated with lipid metabolism, specifically, primary bile acid biosynthesis (impact = 0.07). Furthermore, nitrogen metabolism, which has a function in energy metabolism, was identified as a potential target pathway (*p* < 0.05). These results indicate that six distinct metabolic pathways had a significant influence on VED-affiliated cows.

### 3.4. Validation of the Important DMs and Screening for Novel Biomarkers

**Figure 4:**
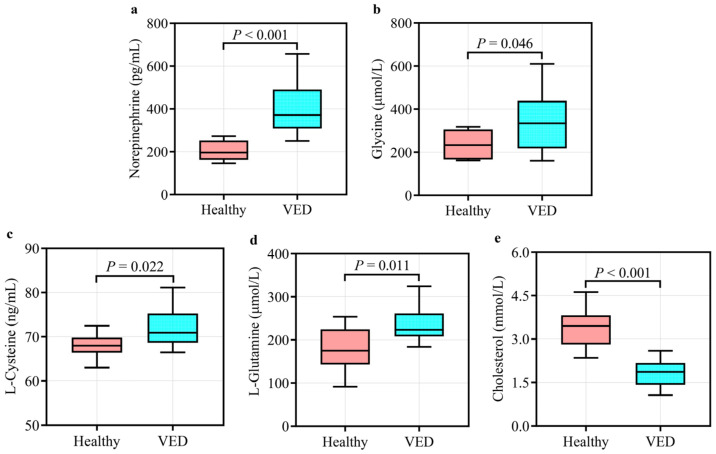
Quantification of the five differential metabolites enriched in six main pathways in the VED (*n* = 10) vs. HC group (*n* = 10): (**a**) norepinephrine; (**b**) glycine; (**c**) L-cysteine; (**d**) L-glutamine; and (**e**) cholesterol.

**Figure 5:**
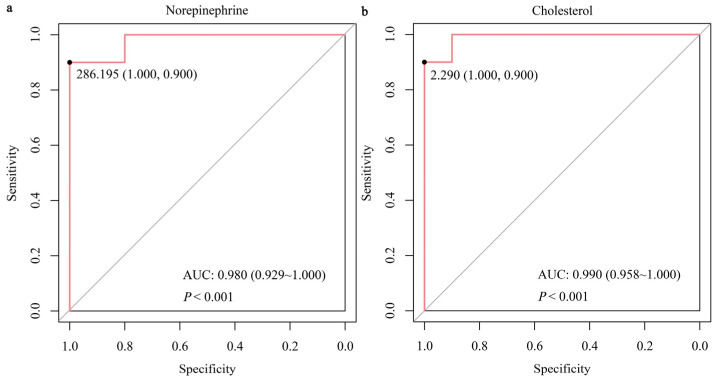
Receiver operating characteristics AUC of norepinephrine (**a**) and cholesterol (**b**) in DCs having VED.

The UPLC-MS system with the MRM mode was employed to verify GC-TOF-MS results. Unlike the HC group, the VED group displayed significantly elevated concentrations of serum norepinephrine, glycine, L-cysteine, and L-glutamine and lower concentrations of serum cholesterol (*p* < 0.05, Figure 4). These outcomes were in line with the GC-TOF-MS analysis. Spearman correlation analysis showed that serum norepinephrine, L-cysteine, and L-glutamine levels had a positive correlation (*p* < 0.05) with VED, while serum cholesterol level was negatively correlated (*p* < 0.01, Table 4). Thereafter, the binary logistic regression analysis manifested that only two of these markers (norepinephrine and cholesterol) exhibited significantly correlation with VED. Here, we utilized ROC curve analysis to assess the accuracy of our examination in identifying useful biomarkers of VED. ROC curve analysis revealed that a norepinephrine level of 286.195 pg/mL effectively distinguished VED from HC cows, exhibiting an area under the curve (AUC) of 0.980 with a sensitivity (SEN) and specificity (SPE) of 90% and 100%, respectively (Figure 5a). The SEN and SPE of cholesterol in diagnosing VED were 90% and 100%, respectively; with a cholesterol cutoff value of 2.290 mmol/L, the AUC was 0.990 (Figure 5b).

## 4. Discussion

This study investigated VED-related metabolic alterations in DCs postpartum through the application of untargeted metabolomics screening. The global non-targeted metabolomics analysis identified 31 potentially VED-related DMs, along with their corresponding metabolic pathways. The results constitute the most comprehensive metabolomics investigation of VED in DCs, possibly facilitating the development of novel diagnostic methods for VED in future clinical veterinary practice.

Throughout EL, most dairy cattle experience a negative energy balance (NEB) period defined by various metabolic alterations, as DMI decreases to insufficient levels to meet the energy requests for maintaining body condition and milk generation [30,31]. Herein, we detected a reduced DMI and BCS in the VED group in contrast to the HCs, suggesting that VED-afflicted cows may experience NEB. Additionally, the decreased BCS observed can be ascribed to reduced DMI [32], which may be a contributing factor to VED [33]. Macrae et al. [34] assessed the prevalence of excessive NEB during the first 20 days of lactation by measuring plasma BHB levels of ≥1.0 mmol/L, NEFA levels of ≥0.7 mmol/L, and glucose levels of ≤3.0 mmol/L. In our study, elevated circulating concentrations of NEFA (nearly 0.79 mmol/L) and BHB (nearly 1.23 mmol/L) indicated that VED-afflicted cows were experiencing a severe NEB state. During the early postpartum phase, severe NEB in dairy cattle is generally marked by the significant mobilization of bodily energy reserves [35]. Thus, the reduced postpartum BCS in VED-afflicted cows indicates an excessive mobilization of adipose tissue [36]. These findings suggest that VED cows experienced increased lipid mobilization during EL due to severe NEB.

Possibly, VED may expedite lipid oxidation. In the VED-afflicted cows, serum ketone body concentrations, such as BHB, increased. The fatty acid β-oxidation within the mitochondria yields BHB [37]. The hypothesis posited that VED enhanced fatty acid β-oxidation, as evidenced by increased BHB levels. The extent of lipid peroxidation and its byproducts is directly proportional to unsaturated fat quantity [38]. Our findings corroborated this, revealing elevated serum levels of unsaturated lipids (elaidic acid) and MDA in VED-afflicted cows. The results offer more proof of the increased oxidation state of cellular conditions attainable through enhanced fatty acid oxidation. This parallels an investigation in which VED led to lipid peroxidation induction [39]. MDA is the primary byproduct of lipid peroxidation [40]; its elevated levels correlate with heightened OxS and degradation regulated by oxidation [41]. Additionally, vitamin E (α-tocopherol) serves as a potent antioxidant, and a reduction in its concentration signifies a drop in T-AOC, which indirectly indicates non-enzymatic antioxidants’ (GSH) levels [42]. In this study, cows with VED exhibit not only high MDA levels but also low T-AOC and GSH levels. Therefore, VED can cause cows to have an OxS response.

A previous metabolomic study has demonstrated that VED is related to alterations in amino acids, lipids, and energy in the serum of DCs [10]. Herein, six pathways linked to amino acid/lipid/energy metabolism were found to be significantly altered in the serum of cows with VED. Five metabolites involved in these pathways were identified, among which norepinephrine, glycine, L-cysteine, and L-glutamine were significantly upregulated, while cholesterol was significantly downregulated.

Norepinephrine is a key neurotransmitter that regulates stress responses and neural activity [43]. Elevated norepinephrine levels typically indicate metabolic stress or physiological challenges. In the VED group, higher norepinephrine levels indicate the metabolic burden and temporary immunosuppression experienced by DCs during the periparturient period. VED weakens antioxidant defenses, resulting in free radical accumulation and heightened OxS [3]. This OxS may activate the sympathetic nervous system, further increasing norepinephrine secretion [44]. Glycine is a fundamental amino acid that has a crucial function in protein synthesis, antioxidant defense, and redox homeostasis [45]. In the VED group, the elevated glycine levels may indicate that DCs are enhancing glycine synthesis to mitigate OxS and improve T-AOC. This process is likely closely associated with glycine’s essential function in one-carbon metabolism (folate cycle) [46]. Moreover, as a key precursor for GSH synthesis, the increased glycine concentration may reflect a heightened demand for GSH production in response to OxS, thereby further supporting the antioxidant defense system [47]. As a sulfur-containing amino acid and direct GSH precursor, L-cysteine is pivotal in thiol metabolism and antioxidant defense [48]. In the VED group, higher L-cysteine levels likely indicate increased demand for thiol metabolism and GSH synthesis to counter OxS. Additionally, the accelerated depletion of GSH caused by exacerbated OxS in VED may further stimulate metabolic pathways to increase L-cysteine availability. L-glutamine is a primary energy source for intestinal epithelial cells and has a critical function in supporting immune function and maintaining intestinal barrier integrity [49]. The elevated concentrations of L-glutamine in the VED group may compensate for energy deficits and enhance mucosal immunity, which is particularly crucial during the immunosuppressive periparturient period. Furthermore, L-glutamine contributes to GSH synthesis through the glutamate cycle, thereby further supporting antioxidant defense [50]. This dual function highlights its importance in alleviating stress related to VED. Cholesterol is an essential element of cell membranes and a precursor for hormone synthesis [51]. Fukui et al. [52] reported that VED in mice causes an elevation in serum cholesterol levels. In contrast to their findings, this investigation detected a reduction in cholesterol levels in VED-afflicted cows, suggesting potential dysregulation of lipid metabolism. This divergence may be ascribed to species-specific variations in lipid homeostasis and metabolic adaptations to OxS. Specifically, VED may impair liver function, suppress cholesterol synthesis, or disrupt cholesterol transport, ultimately leading to decreased cholesterol concentrations. This reduction could negatively impact cell membrane stability and hormone synthesis, further exacerbating metabolic disturbances in DCs. In summary, these outcomes indicate that the identified metabolites could act as potential biomarkers for monitoring DC health and provide valuable insights for targeted clinical interventions.

In this study, the combination of untargeted and targeted metabolomics effectively demonstrated their complementary advantages in revealing metabolic alterations induced by VED. The identification of possibly critical metabolic pathways was made possible by untargeted metabolomics, which has the ability to comprehensively and impartially explore global metabolic alterations [53]. Meanwhile, targeted metabolomics enhanced data reliability and deepened biological interpretation through the precise quantification and validation of specific metabolites [54]. This methodological integration not only improved the accuracy of metabolic profiling but also provided a more comprehensive perspective for understanding the complex metabolic regulatory mechanisms associated with VED. Zhao et al. [55] employed the integrated approach of untargeted and targeted metabolomics to explain the potential mechanisms of lipid mobilization in cows during the early TP. The integration of these two methods significantly enhanced the robustness of metabolomics data and deepened the interpretation of its biological significance. Future research should more extensively adopt this combined strategy to comprehensively elucidate VED-related metabolic disorders, particularly focusing on the mechanisms in key areas such as OxS lipid metabolism and nutritional deficiencies. These efforts will provide scientific evidence for DC health monitoring and targeted interventions.

As mentioned in the introduction, the development of sensitive biomarkers for monitoring the status of VED in DCs continues to be a challenge, impeding progress in the field. Our previous study reported the first comprehensive serum proton nuclear magnetic resonance metabolomics profile of cows with VED and highlighted the importance of targeted metabolomics in conducting quantitative studies of potential biomarkers [10]. Herein, we conducted quantitative targeted metabolomics of the five serum DMs (norepinephrine, glycine, L-cysteine, L-glutamine, and cholesterol) enriched in six main pathways and discovered that these metabolites, particularly norepinephrine and cholesterol, could serve as valuable early indicators of VED in DCs. ROC curve analysis showed that norepinephrine levels of 286.195 pg/mL yielded an AUC of 0.980, with a SEN and SPE of 90% and 100%, respectively, in distinguishing VED cows from HCs. Similarly, cholesterol levels provided excellent diagnostic potential, with a SEN and SPE of 90% and 100%, respectively, at a threshold value of 2.290 mmol/L and an AUC of 0.990. These results emphasize the importance of these two metabolites in diagnosing VED, offering high diagnostic accuracy and potential for early detection. Currently, the diagnosis of VED primarily relies on plasma vitamin E levels, with a threshold of <3 μg/mL [1,3,9,10]. Traditionally, LC-MS is the gold standard method for measuring circulating vitamin E concentrations [26]. However, this method is expensive, time-consuming, and often detects the VED only in its later stages. In contrast, the use of norepinephrine and cholesterol as biomarkers offers a more cost-effective and timely alternative for early diagnosis, enabling intervention before significant clinical symptoms appear. Moreover, the high SEN and SPE of these biomarkers provide a reliable approach for monitoring cows at risk of VED, potentially preventing disease progression. In summary, integrating norepinephrine and cholesterol into VED diagnostic practices could significantly enhance early detection. These findings further emphasize the critical role of metabolic biomarkers in veterinary diagnostics and offer new strategies for early intervention, ultimately improving cow health and productivity.

The current study identifies norepinephrine and cholesterol as potential diagnostic biomarkers for VED in DCs; several methodological limitations warrant careful interpretation. While these biomarkers demonstrate high diagnostic accuracy in our cohort, their non-specific nature raises concerns. Elevated norepinephrine may reflect generalized stress responses rather than VED-specific pathways, and cholesterol alterations could arise from diverse metabolic disruptions (e.g., hepatic dysfunction, energy imbalance, or lipid mobilization disorders), limiting clinical SPE for VED. Direct measurement of serum α-tocopherol remains the gold standard, and the proposed biomarkers may serve best as complementary tools rather than standalone diagnostics in bovine clinical settings. A limited number of enrolled cows (*n* = 27) and case-control design inherently inflate SEN and SPE by minimizing spectrum bias, as evidenced by the 100% SPE—an improbable result in real-world herds with overlapping pathologies. Furthermore, the absence of predefined sample size justification and ambiguous classification criteria (e.g., exclusion of cows with α-tocopherol levels between 3–4 μg/mL) introduce selection bias and reduce external validity. Future validation in larger, prospectively enrolled herds using cross-sectional or longitudinal designs is critical to assess biomarker performance in clinically heterogeneous herds. Notably, biomarker utility must be evaluated against confounding conditions prevalent in transition cows to confirm SPE. For instance, cholesterol levels fluctuate markedly during negative energy balance, potentially masking VED-specific signals. Acknowledging these limitations, our results highlight novel metabolic perturbations in VED-afflicted cows but underscore the need for rigorous validation before clinical translation.

## 5. Conclusions

In conclusion, the application of both untargeted and targeted metabolomics revealed significant metabolic alterations associated with VED, particularly amino acid/lipid/energy metabolism pathways. The ROC curve analysis demonstrated that norepinephrine and cholesterol levels exhibit high diagnostic accuracy, with an AUC of 0.980 and 0.990, respectively, along with a SEN and SPE of 90% and 100%, respectively. These outcomes indicate that norepinephrine and cholesterol have the potential to act as reliable indicators for diagnosing VED in DCs, providing a more cost-effective and timely alternative to the traditional method of measuring plasma vitamin E levels. The integration of these biomarkers into diagnostic practices has the potential to facilitate early intervention and improve disease management, which could contribute to enhanced DC health and productivity. Further investigation is required to evaluate the underlying mechanisms and refine diagnostic protocols for VED.

## Figures and Tables

**Table 1:** Background characteristics and serum biochemical profiles.

Parameters	Healthy (*n* = 14)	VED (*n* = 13)	*p*-Value
Age	3.55 ± 0.95	3.79 ± 0.81	0.488
Parity	2.73 ± 0.88	2.60 ± 1.05	0.730
BCS	3.43 ± 0.41	3.06 ± 0.31	0.014
Milk yield (kg/d)	38.37 ± 7.97	36.29 ± 7.66	0.496
DMI (kg/d)	16.52 ± 0.37	15.46 ± 0.72	<0.001
α-Tocopherol (μg/mL)	6.73 ± 0.84	2.12 ± 0.66	<0.001
NEFA (mmol/L)	0.65 ± 0.18	0.79 ± 0.22	0.081
BHB (mmol/L)	0.98 ± 0.36	1.23 ± 0.29	0.059
Glucose (mmol/L)	7.67 ± 2.03	6.42 ± 2.05	0.124
T-AOC (mmol/L)	0.38 ± 0.05	0.29 ± 0.04	<0.001
SOD (U/mL)	153.09 ± 16.24	145.06 ± 20.68	0.271
Catalase (U/mL)	20.98 ± 1.34	22.02 ± 1.49	0.068
GSH-Px (U/mL)	128.55 ± 16.54	119.42 ± 37.28	0.425
GSH (μmol/L)	83.22 ± 20.86	61.84 ± 23.82	0.020
MDA (mmol/mL)	2.67 ± 0.67	3.98 ± 0.72	<0.001
•OH (U/mL)	613.64 ± 69.81	653.12 ± 50.09	0.106

VED = vitamin E deficiency, BCS = body condition score, DMI = dry matter intake, NEFA = non-esterified fatty acids, BHB = β-hydroxybutyrate, T-AOC = total antioxidant capacity, SOD = superoxide dismutase, GSH-Px = glutathione peroxidase, GSH = glutathione, MDA = malondialdehyde, •OH = hydroxyl radical.

**Table 2:** Serum DMs of both groups screened with untargeted GC-TOF-MS.

No.	KEGG ID	Metabolites	RT (min)	VIP	*p*-Value	FC	VED vs. Healthy
1	C00547	Noradrenaline	12.01	1.60	0.004	4.30	Up
2	C00178	Thymine	7.84	1.15	<0.001	4.26	Up
3	C00584	Prostaglandin E2	14.78	1.88	0.020	4.18	Up
4	C07326	1,5-Anhydroglucitol	10.48	2.46	0.006	3.26	Up
5	C00037	Glycine	7.19	2.85	<0.001	2.97	Up
6	C00451	D-threo-Isocitric acid	10.38	1.76	0.009	2.93	Up
7	—	Diglycerol	10.05	2.06	0.014	2.50	Up
8	C00476	Lyxose	9.48	1.87	0.013	2.37	Up
9	C06555	Biuret	10.04	2.64	<0.001	2.31	Up
10	C01712	Elaidic acid	12.30	1.86	0.033	1.97	Up
11	C01733	Racemethionine	8.61	1.57	0.013	1.87	Up
12	C00805	Salicylic acid	8.58	1.21	0.016	1.83	Up
13	C00085	D-Fructose-6-phosphate	12.71	2.22	0.015	1.74	Up
14	C00097	L-Cysteine	8.81	1.61	0.009	1.62	Up
15	C02057	Phenylalanine	9.29	1.60	0.034	1.49	Up
16	C01877	4-Oxoproline	8.65	1.72	0.030	1.43	Up
17	C01073	N-Acetyl-beta-alanine	7.82	1.50	0.029	1.38	Up
18	C01571	Capric acid	8.23	1.61	0.028	1.37	Up
19	—	Adipamide	9.56	1.49	0.039	1.36	Up
20	C00064	L-Glutamine	8.74	1.48	0.040	1.34	Up
21	C00093	D-Glycerol 1-phosphate	9.96	2.03	0.026	0.65	Down
22	C00665	D-Fructose 2,6-biphosphate	12.21	1.87	0.003	0.64	Down
23	C00392	Mannitol	10.91	1.28	0.047	0.62	Down
24	—	Allylmalonic acid	7.41	1.24	0.007	0.51	Down
25	C00187	Cholesterol	16.77	2.53	<0.001	0.48	Down
26	C06730	4-Methylcatechol	7.78	1.73	0.018	0.46	Down
27	C02477	alpha-Tocopherol	16.53	2.29	0.011	0.40	Down
28	C00180	Benzoic acid	6.79	2.01	0.033	0.39	Down
29	C02591	Sucrose-6-phosphate	15.75	1.96	0.004	0.30	Down
30	—	Methyl phosphate	6.26	3.08	<0.001	0.25	Down
31	—	Hesperitin	15.63	2.00	0.001	0.17	Down

VED = vitamin E deficiency, GC-TOF-MS = gas chromatography tandem time-of-flight mass spectrometry, KEGG = Kyoto Encyclopedia of Genes and Genomes, RT = retention time, VIP = variable importance in projection, FC = fold change.

**Table 3:** Main pathways affected in VED vs. HC groups.

No.	Pathway (Metabolism) Name	Total ^a^	Hits ^b^	Raw *p* ^c^	Holm *p* ^d^	−ln(*p*) ^e^	Impact ^f^
1	Nitrogen	9	2	0.007	0.594	4.92	0.00
2	Glycine, serine, and threonine	32	2	0.082	1	2.50	0.29
3	Alanine, aspartate, and glutamate	23	1	0.297	1	1.21	0.13
4	Cysteine and methionine	28	1	0.350	1	1.05	0.13
5	Tyrosine	42	1	0.477	1	0.74	0.11
6	Primary bile acid biosynthesis	46	2	0.151	1	1.89	0.07

^a^ Compound total number in the pathway. ^b^ Corresponding number of metabolites in one pathway. ^c^ Original *p* value from enrichment testing. ^d^ *p* value adjusted using the Holm-Bonferroni method. ^e^ Negative logarithm base e of the *p* value. ^f^ Pathway influence value from topology analysis.

**Table 4:** Correlation between five target metabolites and VED in DCs.

Parameters	Mean (*n* = 20)	SD	*R*-Value	*p*-Value
Norepinephrine (pg/mL)	306.39	140.75	0.832 **	<0.001
Glycine (μmol/L)	285.26	119.31	0.416	0.068
L-Cysteine (ng/mL)	70.00	4.31	0.503 *	0.024
L-Glutamine (μmol/L)	207.76	55.03	0.538 *	0.014
Cholesterol (mmol/L)	2.61	0.99	−0.850 **	<0.001

*R* is the Spearman rank correlation coefficient. *R* > 0: positive correlation; *R* < 0: negative correlation. * Significant correlation with VED (*p* < 0.05); ** extremely significant correlation with VED (*p* < 0.01).

## Data Availability

Available upon request.

## Supplementary Materials

The following supporting information can be downloaded at: https://www.mdpi.com/article/10.3390/ani15091333/s1, Figure S1: Total ion chromatogram of 4 QC samples; Table S1: Ingredient and chemical composition of the early-lactation TMR diet for 27 multiparous Holstein dairy cows.
