# Supplementary material for: Serum Norepinephrine and Cholesterol Concentrations as Novel Diagnostic Biomarkers for Vitamin E Deficiency in Holstein Cows
Source: Animals (Basel). 2025 May 6;15(9):1333. doi: 10.3390/ani15091333 (PMC12070827; doi:10.3390/ani15091333)
Supplement: Supplementary file 1 [file animals-15-01333-s001.zip › animals-3569967-supplementary.pdf]

Serum Norepinephrine and Cholesterol Concentrations as Novel Diagnostic Biomarkers for Vitamin E Deficiency in Holstein Cows

Journal name: Animals

SUPPLEMENTARY TABLE S1 Ingredient and chemical composition of the early-lactation TMR diet for 27 multiparous Holstein dairy cows

**Ingredient**	**Content (g/kg DM)**
Corn silage	250
Alfalfa hay	120
Oat hay	130
Corn grain, ground	251
Wheat bran	55
Soybean meal	106
Rapeseed meal	25
Cottonseed cake	45
Calcium carbonate	5
Salt	5
Calcium phosphate	3
Mineral and vitamin premix[1]	5
Chemical composition	
Organic matter	919.3
CP	176.4
Ether extract	31.3
NDF	353.1
ADF	191.0
Non-fiber carbohydrate[2]	358.7
Ca	8.3
P	4.2

[1]Contained per kg premix: 20,000 mg Fe, 1,600 mg Cu, 8,000 mg Mn, 7,500 mg Zn, 120 mg I, 20 mg Co, 820,000 IU vitamin A, 300,000 IU vitamin D, and 10,000 IU vitamin E.

[2]Non-fiber carbohydrate, calculated by 1000 – (CP + NDF + Fat + Ash).

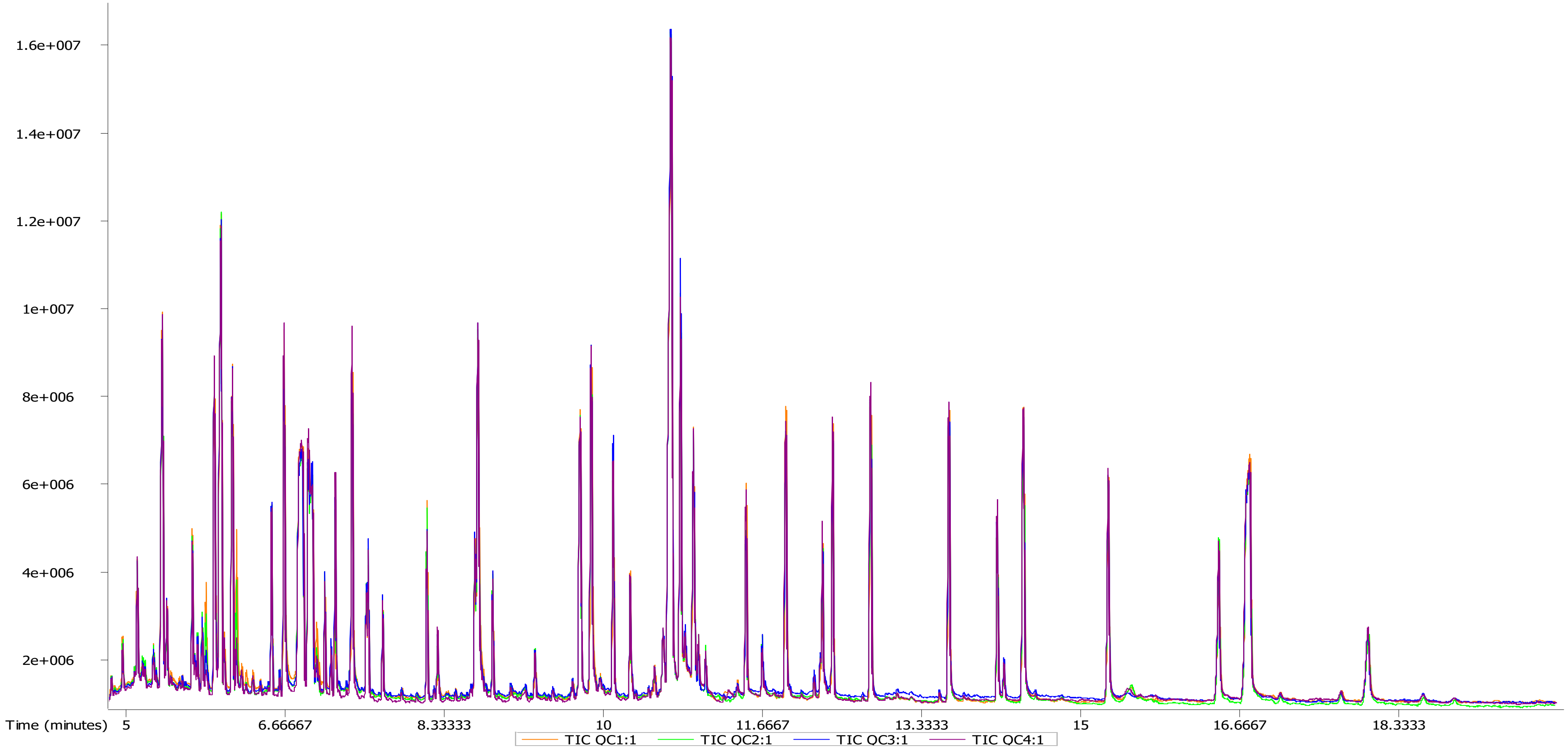

SUPPLEMENTAL FIGURE S1 Total ion chromatogram of 4 QC samples
